# Current role of perampanel in pediatric epilepsy

**DOI:** 10.1186/s13052-017-0368-6

**Published:** 2017-06-02

**Authors:** Paola De Liso, Romina Moavero, Giangennaro Coppola, Paolo Curatolo, Raffaella Cusmai, Giovambattista De Sarro, Emilio Franzoni, Federico Vigevano, Alberto Verrotti

**Affiliations:** 1 Child Neurology Unit, Department of Neuroscience and Neurorehabilitation, “Bambino Gesù” Children’s Hospital, IRCCS, Rome, Piazza S. Onofrio 4, 00165 Rome, Italy; 2 Child Neurology and Psychiatry Unit, Systems Medicine Department, Tor Vergata University of Rome, Via Montpellier, 1, 00137 Rome, Italy; 3 Child and Adolescent Neuropsychiatry, Department of Medicine and Surgery, University of Salerno, Salerno, Italy; 4 Department of Science of Health, Clinical Pharmacological Unit, University “Magna Graecia” of Catanzaro, Catanzaro, Italy; 5 Child Neurology and Psychiatry Unit, University of Bologna, Bologna, Italy; 6 Department of Pediatrics, University of L’Aquila, L’Aquila, Italy

**Keywords:** Perampanel, Pediatric epilepsy, AMPA, Antiepileptic drugs, Tolerability, Efficacy

## Abstract

Perampanel is among the latest AEDs approved, indicated for the treatment of partial-onset seizures with or without secondary generalization, and for primary generalized tonic-clonic seizures, in patients aged 12 years and older. This paper summarizes the clinical recommendations on the current role of perampanel in the treatment of pediatric epilepsies and future directions for research. The optimal dosage should be comprised between 4 and 12 mg/day, with 8 mg/day being the most common dosage used. The rate and severity of adverse events, including psychiatric symptoms, can be decreased by starting at low doses, and titrating slowly. Overall, perampanel presents an acceptable risk/benefit ratio, but special caution should be made to the risk of seizure aggravation and behavioral problems. The favorable cognitive profile, the ease of use of the titration scheme and the once-daily formulation offer advantage over other AEDs and make this drug particularly suitable for adolescent population. Perampanel is a welcome addition to the armamentarium of the existing AEDs, as it represents a new approach in the management of epilepsy, with a novel mechanism of action and a potential to have a considerable impact on the treatment of adolescents with epilepsy.

## Background

Pediatric epilepsy is a common condition, with a prevalence of about 3.2-5.5/1000 in developed countries and an incidence ranging from 41 to 187 per 100.000 [1]. Despite the introduction of new antiepileptic drugs (AEDs) in the last decade a significant proportion of patients still continue to experience seizures [2].

Perampanel is among the latest AEDs approved in more than 45 countries, including United States and Europe. It is a highly selective ionotropic α- amino-3-hydroxy-5-methyl-4-isoxazolepropionic acid (AMPA) glutamate receptor antagonist, indicated for the treatment of partial-onset seizures with or without secondary generalization, and for primary generalized tonic-clonic seizures, in patients aged 12 years and older [3, 4].

This paper summarizes the clinical recommendations on the current role of perampanel in the treatment of pediatric epilepsies and future directions for research.

## Mechanism of action

Perampanel has a novel mechanism of action, since it was developed specifically to target AMPA receptors, that play a fundamental role in fast excitatory synaptic transmission [4].

AMPA receptors may have a significant role in the pathophysiology of epilepsy: not only the expression of seizures but also the progression of epilepsy [5]. These receptors are the most abundant ionotropic glutamate receptors in the mammalian brain. They are localized at excitatory synapses, post-synaptically. AMPA is the main receptor mediating rapid effects of glutamate and underlies the fast component of excitatory post-synaptic potential (EPSP) [6]. AMPA receptor exists in two states, closed, inactive state and open, active state. The AMPA receptors have two external competitive binding sites and two internal non-competitive binding sites. The receptor’s ion channel allows influx of sodium ions and sometimes calcium ions into the neuron [5].

Competitive antagonists of glutamate may be displaced by high levels of glutamate, while non-competitive AMPA receptor antagonists are not displaced by glutamate because they have a different binding site. Receptor antagonism is maintained and the channel remains closed. Perampanel is a non-competitive, selective AMPA receptor antagonist, and it has a wide therapeutic time window [4, 7].

## Pharmacokinetics and drug interactions

Perampanel is rapidly and completely absorbed from gastrointestinal tract after oral administration, it has high oral bioavailability and dose-proportional kinetics. The pharmacokinetics of perampanel are linear and predictable over the clinically relevant dose range (2-12 mg). Peak plasma concentrations are observed 15 min to 2 h after administration [8]. The drug is highly protein bound to albumin and α1-acid glycoprotein (95%-96%), but it has shown good penetrability of the blood-brain barrier. The drug is extensively metabolized (>90%) in the liver to various pharmacologically inactive metabolites. It undergoes oxidative metabolism, primarily via CYP3A4/5, followed by glucuronidation. The terminal half-life (t½) in humans is 105 h; however, in the presence of a strong CYP3A4 inducer (such as carbamazepine, oxcarbazepine and phenytoin), the t½ can be reduced. Topiramate was also able to increase perampanel clearance but to a lesser extent (22.8%) and to mild decrease mean perampanel AUC values by 20% [9]. In sum, perampanel is a selective, centrally acting, negative allosteric modulator of AMPA receptors with good oral bioavailability and favorable pharmacokinetic properties [10, 11]. A population PK analysis of 152 adolescents aged 12-17 years with refractory partial-onset seizures confirmed that pharmacokinetics of perampanel is similar in adolescents and adults [12]. Perampanel has minimal propensity to cause pharmacokinetic interactions. Perampanel decreased oxcarbazepine clearance (by 26%) and increased oxcarbazepine plasma concentrations (by 35%). The clinical relevance of this interaction is difficult to ascertain because the therapeutics of oxcarbazepine are dependent on its pharmacologically active metabolite (10-hydroxycarbazepine), which was not quantified.Efficacy.

### Focal seizures

The strongest data on efficacy of perampanel as add-on treatment of refractory focal seizures in adolescents emerge from pooled data of three phase III randomized controlled studies (304-305-306) and of the subsequent open-label extension study (307) [13–17]. Patients aged 12 to 17 years treated with perampanel as add-on to 1-3 baseline AEDs represent about 10% (143 patients) of a global population of 1480 subjects with partial-onset seizures. Among the 143 adolescents, 98 were randomized to perampanel, and 45 to placebo; 129 completed a core study, and 124 of these (96.1%) were enrolled in the extension study [17]. By the end of maintenance period adolescents had a 35-36% median decrease of seizure frequency at a daily dose of 8-12 mg; the responder rate was 41-45% [17]. The responder rate at 12 months was 47% considering weeks 40-52 of treatment, being even higher in patients with secondary generalized seizures only (64%).

In a randomized study evaluating behavior, efficacy and safety in adolescents (12-17 years) with inadequately controlled partial-onset seizures, perampanel was added to 1-3 baseline AEDs. Of the randomized patients (2:1 ratio), 85 received perampanel and 48 received placebo. Median reduction in seizure frequency from baseline was 58.0% for perampanel and 24.% for placebo (*p* = 0.079). Seizure freedom was achieved in 23.7% of patients on perampanel compared to 16.3% on placebo; responders were 59% in the perampanel and 37% in the placebo group (*p* = 0.014) [18].

Favourable results have been reported also by a retrospective survey including 36 children with focal seizures (age 2-17 years) and finding a responder rate of 33%, with 3 patients achieving seizure freedom and with a trend for higher efficacy in children of 6 years of age and over [19].

Another observational, retrospective study involved 16 tertiary epilepsy centers and retrospectively collected data on 62 children and adolescents who started treatment with perampanel, with a mean follow-up of 6.6 months [20]. Among all analyzed patients, 39 had focal seizures, 10 of whom obtained a ≥ 75% reduction in seizure frequency. In this work, in contrast with other studies, a better response was observed in patients treated with enzyme-inducers AEDs.

### Generalized seizures

There is only one multicenter, randomized, double-blind, placebo-controlled study performed to evaluate the efficacy and tolerability of perampanel in patients aged 12 years and older with primary generalized tonic clonic seizures in idiopathic generalized epilepsies [21]. A total of 164 patients have been randomized, with a higher responder rate for perampanel group than placebo (64.2% vs 39.5%) and a median reduction in seizure frequency of 76.5% for patients on perampanel and 38.4% for patients on placebo. Unfortunately, the data published do not allow to extrapolate specific data for adolescents.

A recent retrospective, multicenter observational survey included a total of 58 children aged 2-17 years, and among them 12 presented with unclassified generalized seizures, 5 with Lennox-Gastaut syndrome, 3 with West syndrome and 2 with Dravet syndrome [19]. The total responder rate in this subgroup of patients was 27% with two patients achieving seizure freedom, one with Dravet syndrome and the other with unclassified generalized epilepsy [19].

### Special etiologies

An Italian multicenter observational study enrolled a total of 62 patients up to the age of 18 years. Besides patients with focal epilepsies they also included epileptic encephalopathies and epilepsies with both focal and generalized seizures [20]. A separate analysis of efficacy was only made for 6 patients with a definite epileptic syndrome (Dravet syndrome, PCDH19, Lennox-Gastaut and myoclonic astatic epilepsy), and a clinically significant response was observed in half of them [20]. Perampanel efficacy has also been assessed in Lafora disease, in two anecdotal cases (one adult, one adolescent) and in a small series of 10 patients [22–24]. Three of these 10 patients were in the pediatric age range and only one of them presented with some benefit [24].

## Safety profile

The pooled data on efficacy and safety of perampanel in adolescents showed an overall favorable risk–benefit profile without alterations of hematologic and clinical chemistry values, vital signs, mean electrocardiogram parameters or skin photosensitivity [17]. The most common treatment emerging adverse events (TEAEs) observed in ≥10% of adolescents patients were: dizziness (20.4%), somnolence (15.3%), aggression (8.2%), decreased appetite (6.1%), and rhinitis (5.1%). During the extension study dizziness (13.2%), somnolence (11.6%), and aggression (6.6%) were the adverse events most often leading to perampanel interruption/dose adjustment. The discontinuation rate due to TEAEs was 14.9% with a rate of serious adverse events of 14.0%. A lower incidence of TEAEs was achieved with a slower titration rate, and adverse events were usually reversible with perampanel discontinuation or with dose adjustment. Perampanel was associated with a 10% worsening of seizures mainly related to intensity and duration of seizures rather than to their frequency in a retrospective real-life study [20]; in RCTs seizure worsening (defined as an increase in seizure frequency > 50%) was observed in 8-11% of cases versus 13% of placebo [20, 25]. A single study on 24 children treated with perampanel showed that adverse events were more frequent in children older than 12 years than in younger ones [26].

An important note should be made to “serious psychiatric and behavioral reactions” which are listed by FDA as potential adverse effects of perampanel. Some recent real life studies, although performed in adult patients, confirm the common occurrence of these adverse events [27–29]. The pooled analysis deriving from the three phase III trials and open label extension study revealed that among the 143 treated, aggression was a complaint in 8.2% [17]. Extrapolating data for patients in pediatric age from other observational studies, data on psychiatric adverse events of 355 subjects up to 18 years of age are available. Among them, challenging behavior (including aggressiveness, irritability and general behavioral disturbances) has been described in 11% [18–20, 30, 31]. A relationship between daily dose of perampanel and psychiatric adverse events has been described, with challenging behavior being more common for higher doses up to 12 mg [32]. Furthermore, these events are much more frequent in the first 6 weeks of treatment, that is the titration phase in clinical studies, although patients could experience new psychiatric events after the initial titration period [32]. The majority of subjects complaining challenging behavior continued the treatment with perampanel, although some at reduced doses, supporting the notion that these psychiatric events can be manageable [32]. However, discontinuation due to psychiatric adverse events occurred in 2.5% of cases in phase III trials [32]. The data available up to now do not allow us to establish if a previous history of psychiatric illness confers a higher risk for developing psychiatric side effects due to perampanel treatment. Since most clinical trials exclude patients with active psychiatric symptoms, real-world data will provide further information on this issue [18]. The results of a recent retrospective study on 464 subjects (*n* = 21 adolescents) treated with perampanel for 12 months suggest that patients with a history of hyperactivity and personality disorders are more likely to develop psychiatric adverse events than those without [33].

Since many AEDs may cause cognitive side effects, it is important to evaluate the impact of every new AED on cognitive functions. For this purpose, the primary objective of a phase II randomized, double-blind, placebo-controlled study in 133 adolescent patients with uncontrolled focal seizures was to assess the impact of perampanel on cognitive functions through an automated system, the Cognitive Drug Research (CDR) system. After 19 weeks of treatment, changes from baseline in the CDR system global cognition score were similar between patients randomized to perampanel and those who received placebo (*p* = 0.145), therefore it was concluded that perampanel may have a favorable cognitive profile [31].

## Conclusion and current clinical recommendations

Clinical recommendations in this paper are based on literature review and on the clinical expertise of the panel members. The main pros and cons of the use of perampanel in paediatric epilepsies are summarized in Tables 1and 2.

**Table 1:** Presentation of the major studies reporting the efficacy of perampanel in children and adolescents with focal seizures

Authors, year	Study type	N of children/adolescents (age)	Perampanel dosage	Responder rates	Seizure reduction
French et al., 2012	multicenter, double-blind, placebo-controlled trial (study 304)	143^a^ (12-17 y)	8-12 mg/day	2 mg: 4.8%	2 mg: +12.8%
				4 mg: 23.1%	4 mg: 23.9%
				8 mg: 40.9%	8 mg: 34.8%
				12 mg: 45%	12 mg: 35.6%
				placebo: 22.2%	placebo: 18.0%
Krauss et al., 2012	multicenter, double-blind, placebo-controlled trial (study 306)		2-8 mg/day		
French et al., 2013	multicenter, double-blind, placebo-controlled trial (study 305)		8-12 mg/day		
Krauss et al., 2013	Extension study for patients completing the double-blind phase of the three previous phase III trials (study 307)		8-12 mg/day	27.3% to 60.0% in adolescents randomized to placebo and switched to perampanel	35.8% for adolescents switched from placebo to perampanel
				40.9% to 54.8% in adolescents receiving perampanel throughout the study	40.9% in adolescents treated with perampanel in both the core and extension studies
Birò et al., 2015	multicenter, observational, retrospective survey	36^c^ (2-17 y)	2-12 mg/day	33%	NR
Lagae et al., 2016	multicenter, randomized, double-blind, placebo-controlled, parallel-group study	133^b^ (12-17 y)	8-12 mg/day	Perampanel: 59%	Perampanel: 58%
				Placebo: 37%	Placebo: 24%

^a^total number of adolescents randomized (98 perampanel, 45 placebo). Of those, 124 entered the extension phase and therefore received at least 1 dose of perampanel

^b^total number of adolescents randomized (85 perampanel, 48 placebo)

^c^From a total of 58 patients with varius epileptic syndromes and seizure types

NR: not reported

**Table 2:** Main advantages and disadvantages for the use of perampanel in childhood epilepsies

Advantages	Disadvantages
Proved effectiveness in partial onset and primarily generalized seizures Favourable cognitive profile Single daily administration Overall good risk/benefit ratio	Possible interaction with enzyme inducers AEDs Possible psychiatric adverse events Few data for children <12 years

Perampanel is effective in partial-onset seizures as well as in primary generalized ones. Although it is only approved for adjunctive treatment of seizures in adolescents 12 years of age and older, how early in the course of refractory epilepsy adjunctive treatment with perampanel should be initiated is unclear. Furthermore, we still don’t know if there are AEDs for which a combined treatment could be more effective, nor if certain epilepsy syndromes are more likely to respond to perampanel. Overall, perampanel presents an acceptable risk/benefit ratio but special caution should be made to the risk of seizure aggravation and behavioral problems.

Perampanel should be given preferably with non-enzyme inducers AEDs, even with sodium channel blockers, although in a recent study the concomitant medications with AEDs increased the chance to become responders [20]. When co-administered to enzyme inducers in fact, a decrease of about 30% of its blood level must be taken into account. Studies show that the optimal dosage should be comprised between 4 and 12 mg/day, with 8 mg/day being the most common dosage used.

According to the clinical experience of the panel members, the rate and severity of adverse events, including psychiatric symptoms, can be decreased by starting at low doses, and titrating slowly. Furthermore, slow titration could also allow an assessment of clinical efficacy of perampanel at low dosages. The risk of some adverse events, such as dizziness, is lower taking perampanel at bedtime. The favorable cognitive profile, ease of use of the titration scheme and once-daily formulation offer advantage over other AEDs and make this drug particularly suitable for adolescent population.

More data are required before any firm conclusions can be drawn with regard to the value of perampanel plasma level monitoring. Since most studies are focused on the antiseizure efficacy of perampanel, as well as on the capture of traditional potential adverse events, standardized cognitive and behavioral tests are needed in pediatric trials. New trials in childhood should investigate the specific efficacy on some specific epileptic syndromes including Lennox-Gastaut, and a study with this aim is already planned (clinicaltrials.gov, NCT02834793).

In conclusion, perampanel is a welcome addition to the armamentarium of the existing AEDs, as it represents a new approach in the management of epilepsy, with a novel mechanism of action and a potential to have a considerable impact on the treatment of adolescents with epilepsy.

## Abbreviations

AEDs: Antiepileptic drugs
AMPA: α-amino-3-hydroxy-5-methyl-4-isoxazolepropionic acid
CDR: Cognitive Drug Research
EPSP: Excitatory post-synaptic potential
FDA: Food and Drug Administration
RCT: Randomized clinical trials
TEAEs: Treatment emerging adverse events
